# Blood–Brain Barrier Dysfunction and the Potential Mechanisms in Chronic Cerebral Hypoperfusion Induced Cognitive Impairment

**DOI:** 10.3389/fncel.2022.870674

**Published:** 2022-06-16

**Authors:** WenQing Xu, Qingke Bai, Qiang Dong, Min Guo, Mei Cui

**Affiliations:** ^1^Department of Neurology, Huashan Hospital, Fudan University, Shanghai, China; ^2^Department of Neurology, Pudong People’s Hospital, Shanghai, China; ^3^State Key Laboratory of Medical Neurobiology and MOE Frontiers Center for Brain Science, Department of Neurology, Huashan Hospital, Fudan University, Shanghai, China

**Keywords:** chronic cerebral hypoperfusion, blood–brain barrier, vascular cognitive impairment and dementia, white matter hyperintensity, normal appearing white matter

## Abstract

Chronic cerebral hypoperfusion (CCH) is a major cause of vascular cognitive impairment and dementia (VCID). Although the underlying mechanisms have not been fully elucidated, the emerging data suggest that blood–brain barrier (BBB) dysfunction is one of the pivotal pathological changes in CCH. BBB dysfunction appears early in CCH, contributing to the deterioration of white matter and the development of cognitive impairment. In this review, we summarize the latest experimental and clinical evidence implicating BBB disruption as a major cause of VCID. We discuss the mechanisms of BBB dysfunction in CCH, focusing on the cell interactions within the BBB, as well as the potential role of *APOE* genotype. In summary, we provide novel insights into the pathophysiological mechanisms underlying BBB dysfunction and the potential clinical benefits of therapeutic interventions targeting BBB in CCH.

## Introduction

As the aging population grows, cognitive impairment, consisting of mild cognitive impairment and dementia, has become a prominent health challenge worldwide. Globally, the number of people afflicted with dementia more than doubled from 20.2 million in 1990 to 43.8 million in 2016, while this figure is estimated to more than triple in 2050 (152.8 million; GBD, 2019, 2022). In China, the prevalence of dementia was 5.60% in 2019 among individuals aged above 65 years (Jia et al., 2020). Vascular cognitive impairment and dementia (VCID), which refers to cognitive impairment and dementia attributed to vascular risks, is deemed as the second leading cause of dementia (van der Flier et al., 2018).

A growing amount of evidence from animal models and epidemiological investigations in humans suggests that chronic cerebral hypoperfusion (CCH) is one of the major pathophysiological hallmarks of cognitive decline (Washida et al., 2019; Zlokovic et al., 2020; Rundek et al., 2021). CCH, despite without a clear definition, is implicated in multiple morbid conditions like heart failure, hypotension, and carotid stenosis (Iturria-Medina et al., 2016; Ciacciarelli et al., 2020), whose relationships to cognitive function have been verified in previous research. For instance, the Whitehall II cohort study demonstrated that adults with atrial fibrillation were associated with accelerated cognitive decline and a higher risk of dementia (Singh-Manoux et al., 2017). Besides, a meta-analysis on the relationship between orthostatic hypotension and cognition also indicated that orthostatic hypotension was associated with an increased risk of dementia and cognitive dysfunction (Peters et al., 2018).

The white matter hyperintensity (WMH) is regarded as the direct manifestation of CCH (Prins and Scheltens, 2015; Prabhakaran, 2019). In a population with high vascular risks, WMH volume was negatively correlated to local perfusion (Jann et al., 2021). Cerebral blood flow (CBF) was lower in WMH (Stewart et al., 2021), and WMH was related to higher risks of cognitive impairment and dementia. Increasing WMH volume started 10.6 years before mild cognitive impairment (MCI) onset on average (Silbert et al., 2012). Moreover, based on a meta-analysis summarizing 23 cross-sectional and 14 longitudinal studies, WMHs exerted a global effect on cognition (Kloppenborg et al., 2014). Taken together, these studies substantiate the pivotal role of CCH in cognition.

The blood–brain barrier (BBB), mainly comprised of cerebral endothelial cells, astrocytes, pericytes, microglia, and basement membrane, is a selective and dynamic interface that separates the central nervous system from peripheral vascularization (Langen et al., 2019; Pandit et al., 2020). By regulating the exchange of substances and cells between neuronal tissues and circulating blood, the BBB maintains the local central nervous environment and homeostasis (Segarra et al., 2021). However, BBB permeability varies under pathological situations, leading to brain dysfunction (Ueno et al., 2016; Pan and Nicolazzo, 2018). For instance, BBB leakage seemed to appear before the onset of hippocampus dystrophy in the early stage of Alzheimer’s disease (AD; Montagne et al., 2019). Besides, BBB impairment and CCH are closely linked to cerebral small vessel disease (CSVD; Wong et al., 2019). Also, BBB failure could predict a poorer functional outcome in patients with CSVD (Wardlaw et al., 2013).

Normal appearing white matter (NAWM) or white matter penumbra, owing to its inclination to evolve into WMH (Maillard et al., 2011), refers to subtle white matter injuries that are undetectable by conventional magnetic resonance imaging (MRI) but are only visible using novel imaging techniques such as diffusion tensor imaging (DTI) and magnetization transfer imaging (MTI; Schmidt et al., 2011). Similar to WHM, lower CBF is also presented in NAWM: the closer to the WMH region, the lower the CBF (Promjunyakul et al., 2015; Wong et al., 2019). Using DCE-MRI, BBB leakage in NAWM was also confirmed in SVD cases (Zhang et al., 2017a). Precisely, leakage rate and area under the leakage curve in the NAWM were found to be proportional to the total CSVD burden in MRI (Li et al., 2018). Furthermore, studies revealed the correlation of NAWM to cognitive performance, which was even better than WMH. In a 2-year follow-up study, higher BBB leakage volume at baseline in NAWM was associated with an increased overall cognitive decline (Kerkhofs et al., 2021). Therefore, BBB disruption is an early change that holds significant prognostic value in CCH-induced cognitive impairment, offering a critical time window for therapeutic intervention.

In conclusion, the clinical evidence indicates that BBB dysfunction is a cardinal factor in CCH-induced VICD.

## Experimental and Clinical Evidence of Blood–Brain Barrier Dysfunction in Chronic Cerebral Hypoperfusion

### Experimental Evidence

#### Blood–Brain Barrier Integrity Detection Based on Structural Components *in vitro*

The intact components of BBB, both at the cell level and the molecular level, ensure its normal functions. Given the ultrastructure, such as the smallest capillaries in the rat brain that are about 4 μm in diameter (Wong et al., 2013), the electron microscope (EM) is applied to directly detect the constituents of BBB. For instance, attenuated capillary density, capillary lumen diameter, increased pericytic degeneration, and the number of endothelial mitochondria were observed under EM in rats that underwent bilateral occlusion of the common carotid arteries (BCAO), concluding that the breakdown of BBB leads to compromised spatial memory (de Wilde et al., 2002). Besides, EM was also used in the observation of large gaps between tight junctions (TJs) of endothelial cells in CCH rats with impaired behavioral performance in the Morris water maze test (Xu et al., 2020).

Furthermore, researchers also developed methods to gage the BBB integrity from a molecular angle. TJs that seal the endothelium preventing BBB leakage (Tietz and Engelhardt, 2015) are widely utilized as markers of BBB permeability. The commonly used TJ molecules include claudins, occludin, and TJ-associated proteins, ZO-1 and ZO-2 (Sweeney et al., 2019). For example, cognitive impairment in CCH rats is combined with BBB disruption in view of reduced expression of occludin and ZO-1 (Lee et al., 2017). Similarly, CCH mice exhibited lower endothelial marker CD31 expression in immunostaining compared to sham controls, suggesting more remarkable BBB damage, which partially explained cognitive decline (Lee et al., 2019). Other markers come from different cell types. CCH-induced mice memory deficits, partially owing to the loss of BBB integrity, were inferred from decreased co-localization of GFAP and AQP4 expression on astrocytic endfeet (Wang et al., 2016b).

#### Blood–Brain Barrier Integrity Detection Based on Functions *in vitro*

The major function of the BBB is to regulate the permeability of molecules and sustain the homeostasis of the brain. Only in cases when the BBB is damaged could make the exchange of large molecules that are supposed to be blocked possible, which is widely used to evaluate the BBB integrity.

After systemic administration, the presence of exogenous tracers within the brain could be used to reflect the BBB integrity qualitatively or quantitatively by investigating the distribution of markers under microscopy or spectrophotometric assays (Kaya and Ahishali, 2011). These compounds are divided into radioisotope and fluorescence-labeled or autofluorescence markers, such as ^14^C-sucrose, ^14^C-mannitol, horseradish peroxidase (HRP), sodium fluorescein, Evans Blue (EB), fluorescein isothiocyanate-dextran, and so forth (Okada et al., 2020; Sun et al., 2021). For instance, more EB extravasation was observed in spontaneously hypertensive rats with severe deficits in spatial memory performance, in contrast to normotensive controls (Choi et al., 2015). Additionally, diffuse leakage of HRP was observed in BCAO rats, judged by the dispersion of the HRP reaction product by microscopy from plasmalemmal vesicles in the endothelial cells to the cytoplasm in endothelial cells and glial cells (Ueno et al., 2002).

### Clinical Evidence

#### Blood–Brain Barrier Integrity Detection Based on Biomarkers in Serum and Cerebral Spinal Fluid

In humans, instead of measuring these protein levels *in situ*, which is rare since brain biopsies are not easily accessible, clinicians turn to search for fluid biomarkers in serum and cerebral spinal fluid (CSF) that come from the degradation of BBB under pathological conditions, such as occludin, S100, claudin-4, ZO-1, fibronectin, matrix metalloproteinases (MMPs), and UCH-L1 (Okada et al., 2020). For example, the elevated level of MMP2 index (MMP_CSF_/albumin_CSF_)/(MMP_blood_/albumin_blood_) is closely aligned with the clinical diagnosis of Binswanger disease, one subtype of VCI (Rosenberg et al., 2015). Besides, S100β, primarily synthesized by the astrocytic endfeet process, was found to be upregulated in serum derived from patients with VICD and correlated to cognitive impairment (Wang et al., 2017). Additionally, upregulation of soluble platelet-derived growth factor receptor-β (sPDGFRβ) in CSF, mainly derived from damaged pericyte, indicated BBB leakage in individuals with AD (Miners et al., 2019), which implicated the potential value of sPDGFRβ as a biomarker of BBB breakdown, but it needs to be further evaluated in CCH.

Alternatively, some molecules in circulating blood can abnormally enter CNS through damaged BBB under morbid conditions, indicating the increased permeability of BBB. The most widely used molecules are albumin and IgG. For example, an increased CSF/serum albumin ratio, which was significantly associated with a higher composite vascular risk score, was identified in patients with MCI, suggesting the possible role of BBB dysfunction in inducing cognitive impairment (Lin et al., 2021). Also, patients diagnosed with Binswanger disease showed higher grading scores for IgG extravasation, namely, worse BBB breakdown, than those without neurological diseases, in both the periventricular and subcortical white matter (Akiguchi et al., 1998).

#### Clinical Imaging

A myriad of novel imaging techniques (Elschot et al., 2021) have been applied in assessing BBB integrity in CSVD, among which the most accepted one is DCE imaging, combined with computed tomography (CT), positron emission tomography (PET)/CT, MRI, or near-infrared spectroscopy (NIRS; Raja et al., 2018; Thrippleton et al., 2019; Sun et al., 2021). For instance, patients with MCI revealed a higher BBB permeability index (i.e., the ratio between late enhancement at the 4th to 5th min to the peak value at 50 s) than those in the control group as measured by DCE-MRI (Wang et al., 2006). Second, dynamic susceptibility contrast (DSC), traditionally considered a perfusion imaging method (Sourbron and Buckley, 2013), showed potential in BBB integrity evaluation by calculating parameters like relative recirculation (Liebner et al., 2018). Specifically, BBB leakage parameters in DSC, representing BBB failure, were significantly correlated with total WML volume even in asymptomatic individuals (Dewey et al., 2021). Besides, arterial spin labeling (ASL) MRI gauges arterially labeled blood spins that are drained into cerebral veins to further determine water extraction fraction (E) and permeability-surface-area product (PS) of BBB (Lin et al., 2018). For example, using water extraction with phase-contrast arterial spin tagging (WEPCAST) MRI, a higher PS value (leakier BBB to water) was implicated in MCI cases compared to normal counterparts (Lin et al., 2021).

## Updated Evidence of Blood–Brain Barrier Dysfunction Mechanisms Involved in Chronic Cerebral Hypoperfusion

**FIGURE 1:**
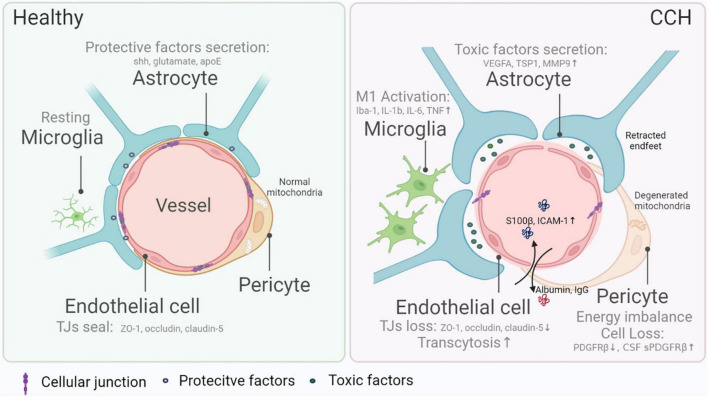
Blood–brain barrier (BBB) damage in chronic cerebral hypoperfusion (CCH). The composition of the BBB mainly includes endothelial cells, pericytes, astrocytes, and microglia, all of which are indispensable in the maintenance of normal BBB integrity and functions. Endothelial cells form a seamless barrier that confines solute exchange between the CNS and the systemic vasculature, attributed to intercellular junction molecules (e.g., ZO-1, occluding, claudin-5), while the loss of barrier in CCH gives rise to BBB leakage and leads to more frequent substance exchange like albumin and IgG entering CNS. Pericytes, wrapping most endothelial cells, protect BBB completeness via nurturing endothelial cells and controlling substance transportation. In CCH, diminishing and malfunction of pericytes cause BBB dysfunction. Reduced pericyte marker PDGFRβ *in situ* and elevated sPDGFRβ in the cerebral spinal fluid are hallmarks of CCH. The function or metabolism of the pericyte is also vital in regulating BBB damage. For example, energy imbalance resulting from mitochondria degeneration causes the malfunction of pericytes in CCH. Furthermore, astrocytes excrete cytokines that might either be protective (e.g., sonic hedgehog, glutamate, and apoE) or toxic (e.g., VEGFA, TSP1, and MMP9). The environmental changes in CCH trigger off harmful factors release and lead to increased BBB permeability. Also, microglia exert dual effects on BBB physiologically, while in CCH, in general, pro-inflammatory subtypes worsen BBB damage.

The BBB is not only comprised of a single layer of endothelial cells that form the interface between the CNS and the periphery entities but also refers to other components such as pericyte, astrocyte, and microglia (Figure 1). Here, we investigate how the BBB is damaged by CCH, focusing on cell interactions and gene hints.

### Endothelial Cell

The endothelial cells within BBB are endowed with unique characteristics that are differentiated from those in the peripheral vasculature (Langen et al., 2019). First, between the brain microvascular endothelial cells (BMEC), TJs physically fill the gap and form a seamless barrier. Next, the highly selective nature of BBB requires a highly arranged expression of transporters within the cell membrane. Third, the BBB physically prevents and regulates transcytosis of solutes through the endothelial cells in physiological milieus (Liebner et al., 2018). Additionally, low leukocyte adhesion molecule (LAM) expression on the endothelium limits the entrance of immune cells into the brain. In CCH, however, endothelial cells are dysregulated. An increase of serum antibodies against the NR2 subunit of the NMDA receptor (NR2ab), probably due to endothelial damage in the early stage of SVD, was confirmed in patients with subjective cognitive impairment (Dobrynina et al., 2021). *In vitro* studies also observed endothelial injury and BBB dysfunction in CCH rats with worse performance in the Morris water maze test (Ueno et al., 2009). The TJ-related protein, ZO-1, was found to be downregulated in CCH mice, targeted by TNFα–miR-501-3p axis (Toyama et al., 2018). Besides, downregulation of major facilitator superfamily domain-containing protein 2a (Mfsd2a) might cause cognitive impairment in CCH rats by promoting transcytosis in endothelial cells and exacerbating BBB breakdown (Qu et al., 2020).

### Pericyte

Encircling a large proportion of endothelial cells in the brain (Armulik et al., 2011), pericytes contribute to the normal function of BBB in multiple ways: supporting endothelial cells (Cheng et al., 2018; Procter et al., 2021), participating in the exchange of substances between CNS and systemic circulation directly (Balabanov and Dore-Duffy, 1998; Ferland-McCollough et al., 2017), begetting neuroinflammation (Rustenhoven et al., 2017), etc. Thus, it is not surprising to monitor the alteration of pericyte number and function in BBB integrity. Around 35–45% decrease in pericyte number in the frontal white matter was confirmed in patients with vascular dementia (VaD) compared to healthy aging controls (Ding et al., 2020). In the postmortem samples of patients with AD, poorer precuneus perfusion was implicated to be associated with attenuated expression of platelet-derived growth factor receptor-β (PDGFRβ; a pericyte marker), indicating pericyte damage and BBB dysfunction (Miners et al., 2018). CSF sPDGFRβ was also found to be elevated in early cognitively impaired patients, independent of Aβ and tau (Nation et al., 2019). In CCH rats, mitochondria degeneration in pericytes was observed under electron microscopy 7 and 14 days after BCAO operation (Ueno et al., 2002), implying an energy imbalance of pericytes. Together, these findings emphasize the importance of pericytes in BBB dysfunction.

### Astrocyte

As the most abundant glial cells in the brain, astrocytes cast a double-faced impact on the completeness of BBB. On the one hand, astrocytes excrete morphogens (sonic hedgehog and retinoic acid), trophic factors (VEGF), gliotransmitters (glutamate), and apolipoprotein E (apoE), among others, to protect BBB. On the other hand, in algetic conditions, the same protective factors could increase BBB permeability (VEGF and glutamate induce junctional damage), or different factors like MMP and nitric oxide, derived from reactive astrocytes, cause damage to BBB (Michinaga and Koyama, 2019; Spampinato et al., 2019). In CCH-induced VaD, lipocalin-2 (LCN2), a neutrophil gelatinase-associated lipocalin, could activate astrocytes to upregulate vascular endothelial growth factor A (VEGFA), which in turn elevated vascular permeability (Kim et al., 2017). Moreover, by manufacturing more exosomal TSP1 (a secretory glycoprotein that mediates cell-cell interactions), which was upregulated in VaD patients’ serum, astrocytes led to BBB disruption (Chen et al., 2020).

### Microglia

Microglias have dual roles on the BBB as either protective or detrimental, reflecting the previously accepted dichotomy: M1, the pro-inflammatory subtype and M2, the anti-inflammatory subtype (Kang et al., 2020; Ronaldson and Davis, 2020). In CCH-induced cognitive impairment models, M1 microglia were found abundantly in the brain parenchyma and perivascular sites. For instance, in CCH rats, activated microglia with enlarged soma and thicker processes were observed in the hippocampus CA1 subarea, together with upregulated cytokines like IL-1b, IL-6, and TNF, which involved BBB breakdown (Hei et al., 2018). Moreover, pro-inflammatory microglia (CD68 positive) were detected at the site of BBB leakage (dextran positive) in angiotensin II (Ang-II) induced hypertension mice with impaired short-term memory (Kerkhofs et al., 2020). On the contrary, on being polarized to the M2 phenotype by activating nuclear factor-like 2 (Nrf2), microglia in the cortex could preserve BBB integrity and improve cognition in CCH rats (Mao et al., 2019), indicating the possibility of regulating the phenotype of microglia in protecting BBB.

### Genetic Clues: *APOE* as a Promising Gene Target

In the CCH entities, the role of *APOE* in BBB breakdown has taken center stage recently. There are three *APOE* isoforms in humans, *APOE*ε*2, APOE*ε*3*, and *APOE*ε*4* (Serrano-Pozo et al., 2021). *APOE*ε*4*, the most prevalent risk factor of sporadic AD (Michaelson, 2014), was recently revealed to be the top overlapping gene between AD and vascular pathology (Lin et al., 2019). *APOE*ε*4* presence could significantly predict the diagnosis of VaD (Stocker et al., 2020), making it one of the strongest risk genes for VaD (Ikram et al., 2017). Furthermore, reducing cerebrovascular reactivity might be an early pathological change in *APOE*ε*4* allele-induced cognitive impairment (Suri et al., 2015). Compared to normal aging, patients bearing *APOE*ε*4* demonstrated a more considerable cerebral blood flow drop (Zhang et al., 2017b). Also, *APOE*ε*4* mutant individuals had a greater decrease in fractional anisotropy in the genu and the splenium of the corpus callosum detected by DTI (Williams et al., 2019). Similarly, after BCAS operation, CBF decline was more evident in apoE-deficient mice than in wild-type controls, the former of which, not surprisingly, showed more severe BBB dysfunction (Lee et al., 2019). Concerning molecular mechanisms, research elucidated the direct impact of apoE on BBB *via* triggering the proinflammatory CypA-NFκB-MMP-9 pathway in pericytes (Bell et al., 2012). In a cohort study of 245 participants, *APOE*ε*4* carriers exhibited more extensive BBB leakage and worse cognitive impairment, independent of AD pathology, of which an activated CypA-MMP9 pathway in CSF was confirmed (Montagne et al., 2020). Thus, targeting *APOE*ε*4* was proved to be a promising therapeutic method in CCH-induced individuals with VICD.

## Potent Therapeutic Interventions Targeting Blood–Brain Barrier in Chronic Cerebral Hypoperfusion

### Blood–Brain Barrier Function Recovery

Given the complex composition of the BBB, potent therapies that target multiple cell components have been raised (summarized in Table 1). For instance, in pericytes, PDGFRβ was found to be elevated by NXP031 (a vitamin C/DNA aptamer complex that boosts vitamin C’s antioxidant efficacy) in VaD mice, which demonstrated better cognitive performance (Lee et al., 2021). Moreover, it was reported that dl-3-n-butylphthalide treatment suppressed astrocyte activation (given by repressed GFAP, TNF-α, and IL-6 expression) in CCH mice, which partially explained the attenuated spatial memory dysfunction (Han et al., 2019). Environmental enrichment (a multitarget rehabilitation method based on early lifestyle modification) partially improved the memory of VCI rats by reversing TJ downregulation caused by CCH (Xu et al., 2020). However, these treatments were all tested in animal models which require further validation in humans.

**TABLE 1:** Therapeutic interventions and the underlying mechanisms targeting blood–brain barrier in chronic cerebral hypoperfusion.

			Target cells			
Therapy	Reference	Model	Endothelial cell	Pericyte	Astrocyte	Microglia
Edaravone	Ueno et al., 2009	BCAO rats	↑eNOS	NA	↓Nitrotyrosine	NA
ASK1 knock out	Toyama et al., 2014	BCAS mice, OGD	↑TJs (occludin, claudin-5)	NA	↓GFAP	NA
Treadmill exercise	Lee et al., 2017	BCAO rats	↑TJs (ZO-1, claudin-5) ↑Microvessel length	NA	NA	NA
MiR-501-3p inhibition	Toyama et al., 2018	BCAS mice	↑TJ (ZO-1)	NA	NA	NA
Histidine	Song et al., 2018	BCAS mice	↑TJ (ZO-1, occludin)	NA	↓Distance to penetrating artery	NA
Dl-3-n-butylphthalide	Han et al., 2019	BCAS mice	↑TJs (occludin, claudin-5)	NA	↓GFAP, ↓MMP-2, ↓MMP-9, ↓TNF-α, ↓IL-6	NA
Sulforaphane	Mao et al., 2019	2VO rats	↑HO-1, ↑TJs (claudin-5, occludin)	NA	↑HO-1	↑HO-1
Triptolide	Yao et al., 2019	BCAO rats	↑TJs (ZO-1, claudin-5)	NA	NA	NA
Environmental enrichment	Xu et al., 2020	2VO rats	↑TJs (ZO-1, claudin-5), ↓Large gap between the TJs	NA	NA	NA
Mfsd2a overexpression	Qu et al., 2020	2VO rats	↑Vesicular transcytosis, unchanged TJs (ZO-1, claudin-5, occludin)	NA	NA	NA
NXP031	Lee et al., 2021	BCAO rats	↑AJ (PECAM-1), ↓Fragmented, longer vessels	↑PDGFRβ	NA	↓Iba-1
C3aR knock out	Bhatia et al., 2022	BCAS mice	↑TJs (ZO-1, claudin-5), ↓Inflammation (↓VCAM1)	NA	NA	↓Iba-1, ↓pSTAT3/STAT3

*ASK1, apoptosis signal-regulating kinase 1; Mfsd2a, major facilitator superfamily domain-containing protein 2a; C3aR, complement C3a Receptor; BCAS, bilateral common carotid arteries stenosis; BCAO, bilateral carotid artery occlusion; OGD, oxygen-glucose deprivation; TJ, tight junction; AJ, adherence junction; NA, not available; PECAM-1, platelet/endothelial cell adhesion molecule 1; HO-1, hemo oxygenases-1; PDGFRβ, platelet-derived growth factor receptor-β; VCAM1, vascular cell adhesion molecule 1; STAT3, signal transducer, and activator of transcription 3.*

### Cell Therapy

Beyond recovering BBB function, an alternate therapeutic intervention is to maintain BBB integrity by transplanting cells. Particularly, by transplanting endothelial progenitor cells (EPCs) into the hippocampus of *APP/PS1* transgenic AD mice, Zhang et al. upregulated TJ expression and repaired BBB, ultimately improving spatial learning and reference memory in the Morris water maze test (Zhang et al., 2018). Similarly, Nakazaki et al. elicited functional recovery in CSVD rats *via* intravenous infusing mesenchymal stem cells (MSC), in which restoration of endothelial cells and pericytes was found in BBB (Nakazaki et al., 2019). Furthermore, preconditioning the CCH rat model with VEGF, a BBB breakdown inducer allowed bone marrow mononuclear cells (BMMNC) to migrate into the brain parenchyma, resulting in better learning capability and memory performance (Wang et al., 2016a). All in all, albeit immature, cell therapy demonstrates great potential in restoring the physiological function of the BBB and preventing or reducing cognitive decline.

## Conclusion

In this review, we stressed the significance of BBB abruption in CCH-induced cognitive deterioration by expounding the findings from the latest research on BBB failure. These results raise awareness and encourage us to pay more attention to the BBB dysfunction in the early stage of CCH and provide us with a new avenue for developing therapies targeting BBB to improve cognitive functions in individuals with CCH.

## Author Contributions

MG, MC, WX, and QB searched data for this review, wrote the manuscript, and contributed substantially to discussions of its content. QD helped with the revision, polishing, and editing of the manuscript before submission. All the authors read, revised, and approved the final manuscript.

## Conflict of Interest

The authors declare that the research was conducted in the absence of any commercial or financial relationships that could be construed as a potential conflict of interest.

## Publisher’s Note

All claims expressed in this article are solely those of the authors and do not necessarily represent those of their affiliated organizations, or those of the publisher, the editors and the reviewers. Any product that may be evaluated in this article, or claim that may be made by its manufacturer, is not guaranteed or endorsed by the publisher.
